# Supplementary material for: TAD evolutionary and functional characterization reveals diversity in mammalian TAD boundary properties and function
Source: Nat Commun. 2023 Dec 7;14:8111. doi: 10.1038/s41467-023-43841-8 (PMC10703881; doi:10.1038/s41467-023-43841-8)
Supplement: Supplementary file 1 — Supplementary Information [file 41467_2023_43841_MOESM1_ESM.pdf]

Supplementary Information

TAD Evolutionary and functional characterization reveals diversity in mammalian TAD boundary properties and function

Mariam Okhovat[1*#], Jake VanCampen[1*], Kimberly A. Nevonen[1], Lana Harshman[2,3], Weiyu Li[2,3], Cora E. Layman[1], Samantha Ward[1], Jarod Herrera[1], Jackson Wells[1], Rory R. Sheng[2,3], Yafei Mao[4,5], Blaise Ndjamen[6], Ana C. Lima[7], Katinka A. Vigh-Conrad[7], Alexandra M. Stendahl[7], Ran Yang[7], Lev Fedorov[8], Ian R. Matthews[9], Sarah A. Easow[9], Dylan K. Chan[9], Taha A. Jan[10], Evan E. Eichler[4,11], Sandra Rugonyi[12], Donald F. Conrad[7,13], Nadav Ahituv[2,3#], Lucia Carbone[1,7, 13,14#]

1. Department of Medicine, Knight Cardiovascular Institute, Oregon Health and Science University, Portland, OR, USA
2. Department of Bioengineering and Therapeutic Sciences, University of California San Francisco, San Francisco, CA, USA
3. Institute for Human Genetics, University of California San Francisco, San Francisco, CA, USA
4. Department of Genome Sciences, University of Washington School of Medicine, Seattle, WA USA
5. Bio-X Institutes, Key Laboratory for the Genetics of Developmental and Neuropsychiatric Disorders, Ministry of Education, Shanghai Jiao Tong University, Shanghai, China (present affiliation)
6. Histology and Light Microscopy Core Facility, Gladstone Institutes, San Francisco, CA, USA
7. Division of Genetics, Oregon National Primate Research Center, Beaverton, OR, USA
8. OHSU Transgenic Mouse Models Core Lab, Oregon Health and Science University, Portland, OR, USA
9. Department of Otolaryngology-Head and Neck Surgery, University of California, San Francisco, CA, USA
10. Department of Otolaryngology-Head and Neck Surgery, Vanderbilt University Medical Center, Nashville, TN, USA
11. Howard Hughes Medical Institute, University of Washington, Seattle, WA 98195, USA
12. Department of Biomedical Engineering, Oregon Health and Science University, Portland, OR, USA
13. Department of Molecular and Medical Genetics, Oregon Health and Science University, Portland, OR, USA
14. Department of Medical Informatics and Clinical Epidemiology, Oregon Health and Science University, Portland, OR, USA

*These authors equally contributed to the project

#Corresponding authors

Correspondence to carbone@ohsu.edu, okhovat@ohsu.edu, nadav.ahituv@ucsf.edu

1. Supplementary Figures

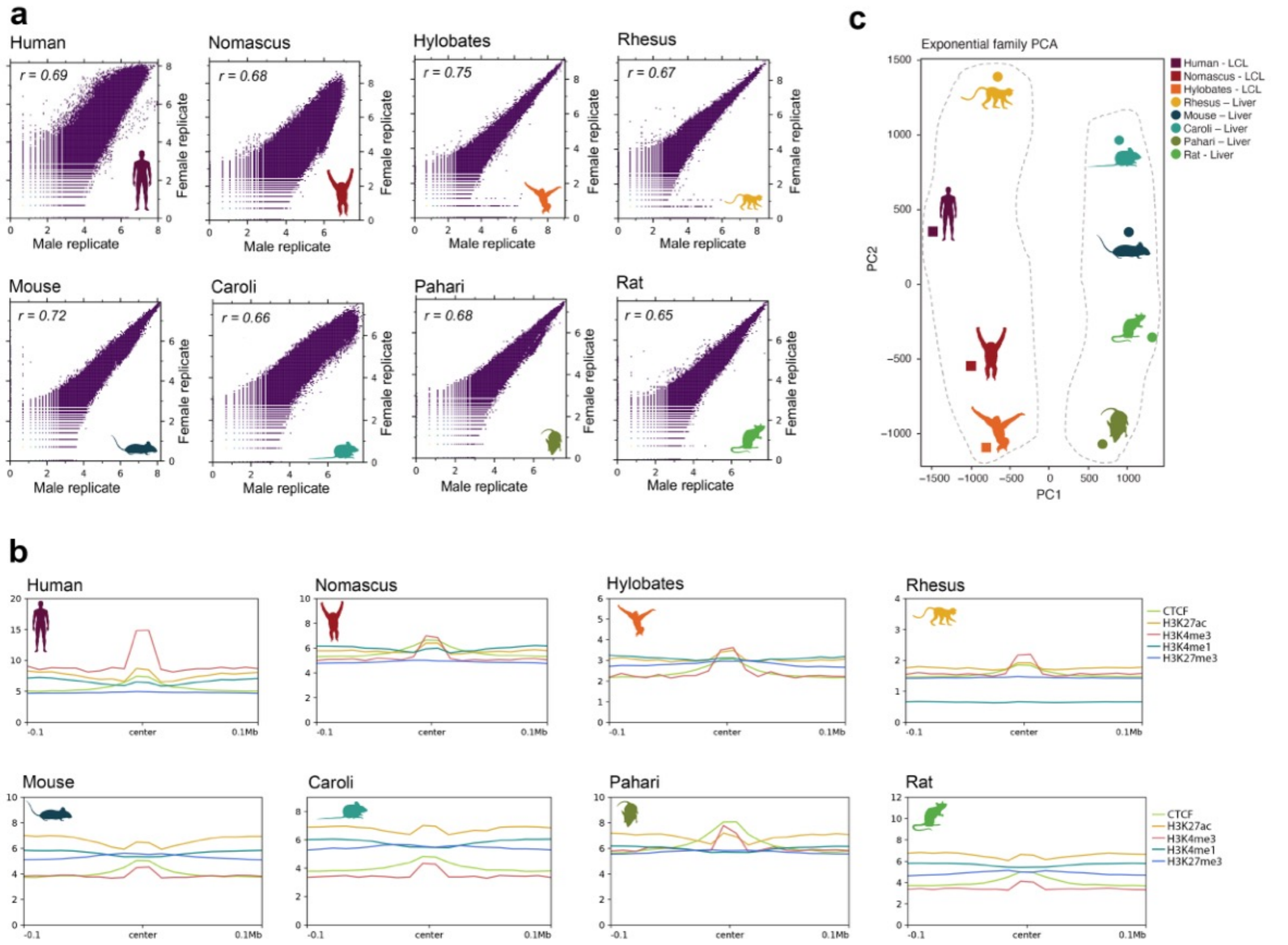

Supplementary Fig. 1: Characterization of TAD boundaries in eight different species. a, Pearson correlation of Hi-C data show correlation between biological replicates. **b,** Plot of the principal components from logarithmic PCA of genome-wide TAD boundary distribution shows separation of samples based on species order (primate vs. rodent) along the first principle (PC1). **c**, Species-specific histone (H3K4me3, H3K4me1, H3K27ac, and H3K27me3) and CTCF ChIP-seq profiles at TAD boundaries show enrichment for active epigenetic marks and depletion of repressive marks across all species. Following artwork licensed and modified from iStock.com/Atlas Studio (human icon), iStock.com/Bullet_Chained (gibbon icons), iStock.com/AlonzoDesign (macaque icon), iStock.com/Nedea (mouse, Caroli, Pahari icons), iStock.com/ ~Userba9fe9ab_931 (rat icon).

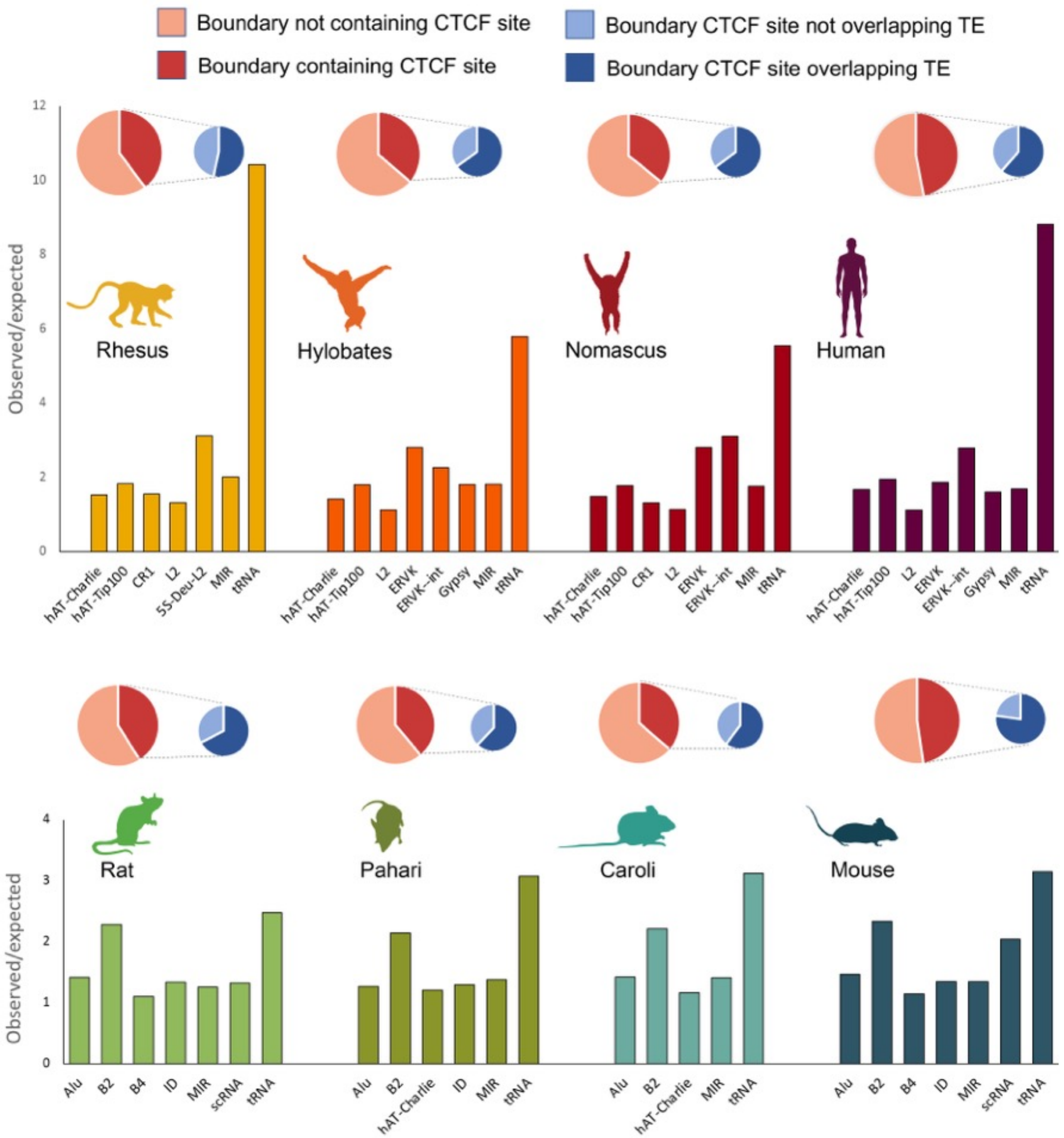

Supplementary Fig. 2: Transposable elements (TE) content at CTCF binding sites in TAD boundaries of each species. Red pie charts show proportion of TAD boundaries containing at least one CTCF binding site in each species, and the blue pie chart demonstrates proportion of TAD boundary CTCF sites that overlap at least one TE. Bar graphs demonstrate observed/expected ratios of TE families significantly enriched ($p<0.05$) at CTCF binding sites in TAD boundaries of all eight species.

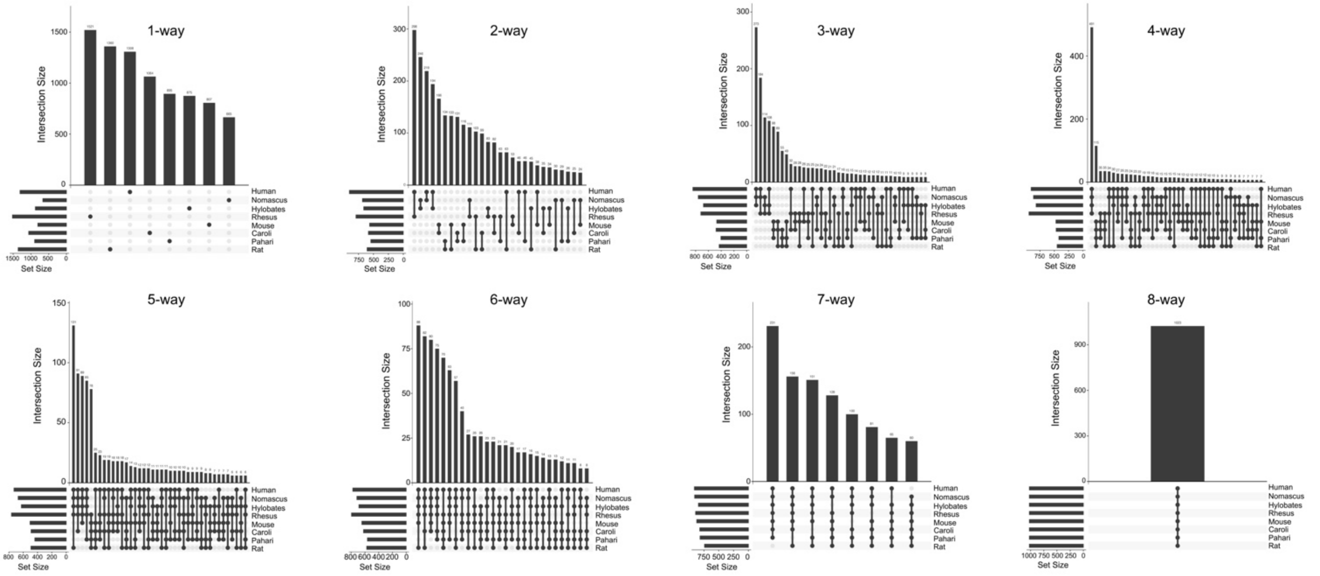

Supplementary Fig. 3 – Upset plots show frequency distribution of cross-species overlap patterns and confirm preferential overlap by species rather than by tissue.

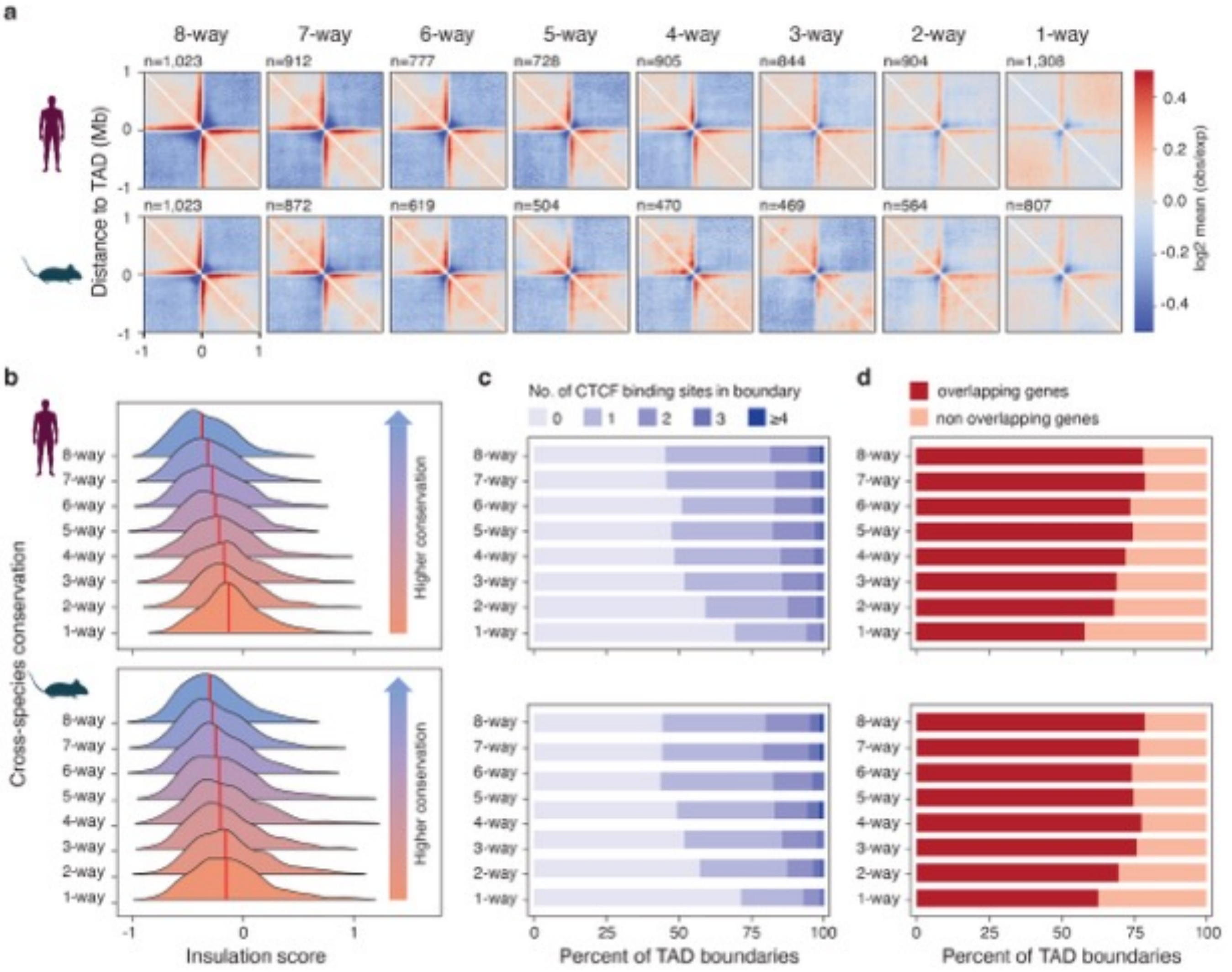

Supplementary Fig. 4: Epigenetic and genetic properties of boundaries vary as a function of their cross-species conservation. **a**, Heatmaps show frequency of genomic interactions (log2 mean observed/expected) across TAD boundaries increase as the TAD boundary conservation decreases from 8- to 1-way conservation, in both human and mouse genomes. **b**, Kernel density plots (median marked with red line) show lower insulation scores (i.e., stronger TAD separation) as boundary conservation increases. **c**, Percent TAD boundaries overlapping CTCF binding sites and **d**, genes are positively associated with the number of species sharing the boundary

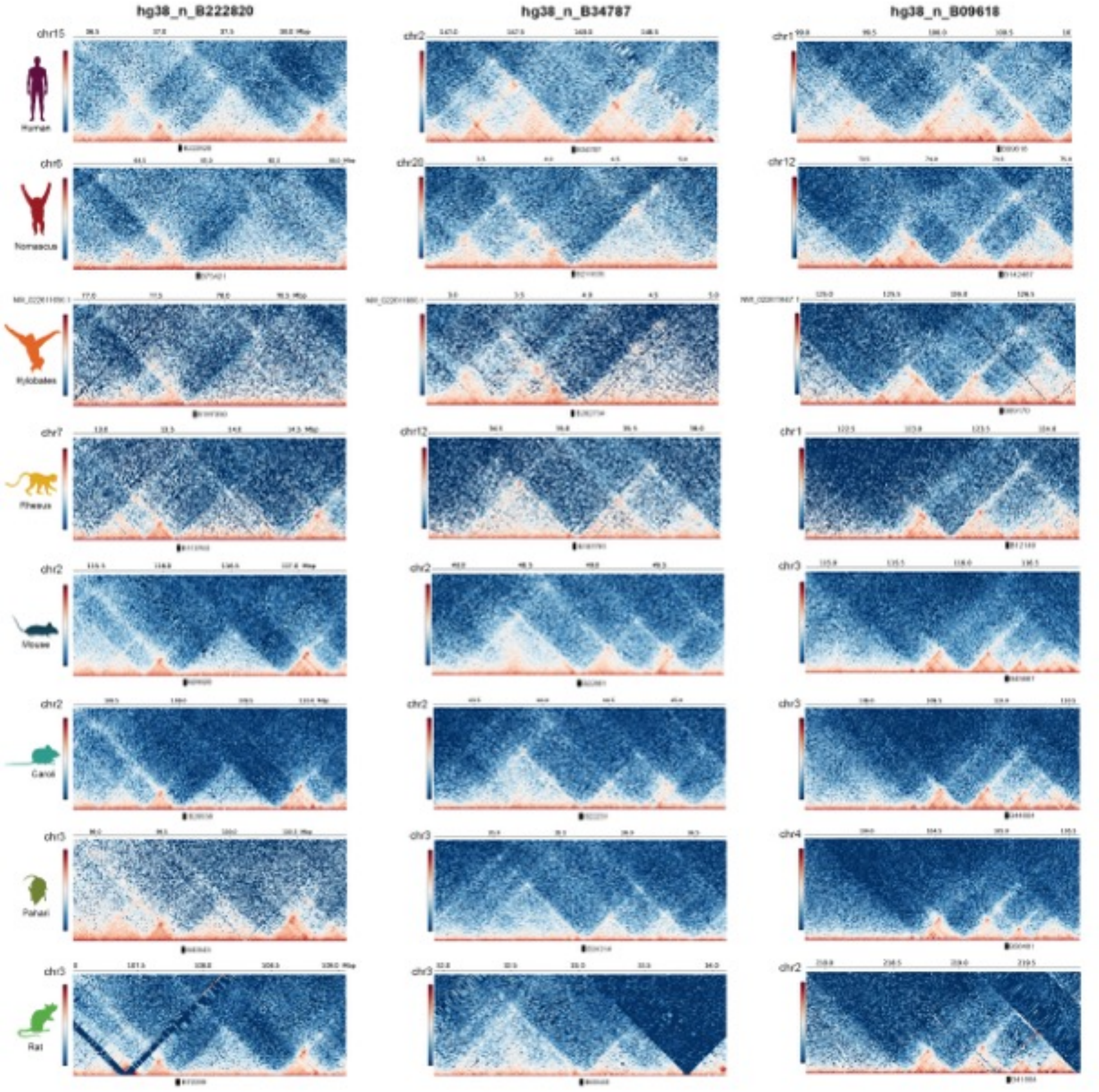

Supplementary Fig. 5: Examples of ultraconserved TAD boundaries. Hi-C matrices for orthologous genomic regions in the different species included in the study show high similarity in genome conformation at ultraconserved TAD boundaries.

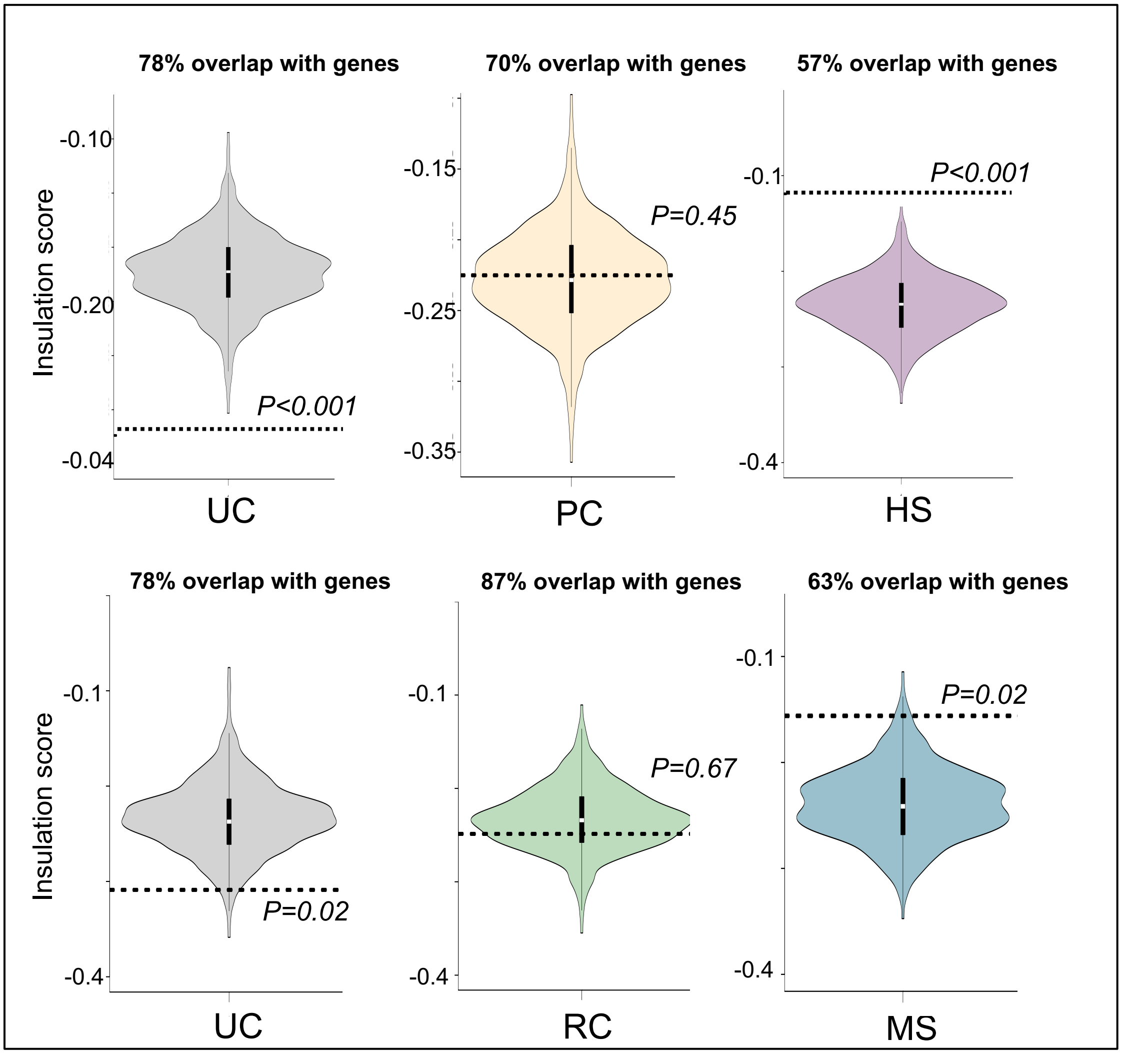

Supplementary Fig. 6 – Violin plots show kernel density of median insulation score in randomly permuted TAD boundaries in human (top row) and mouse (bottom row) genomes. Percentage of TAD boundaries overlapping genes are reported above each violin plot. The observed median insulation score values in each evolutionary group is shown with dashed line, along with the empirical p-value. Abbreviations are as follows: UC= Ultraconserved, PC= Primate-Conserved, HS= Human-Specific, RC= Rodent-Conserved, MS= Mouse-Specific.

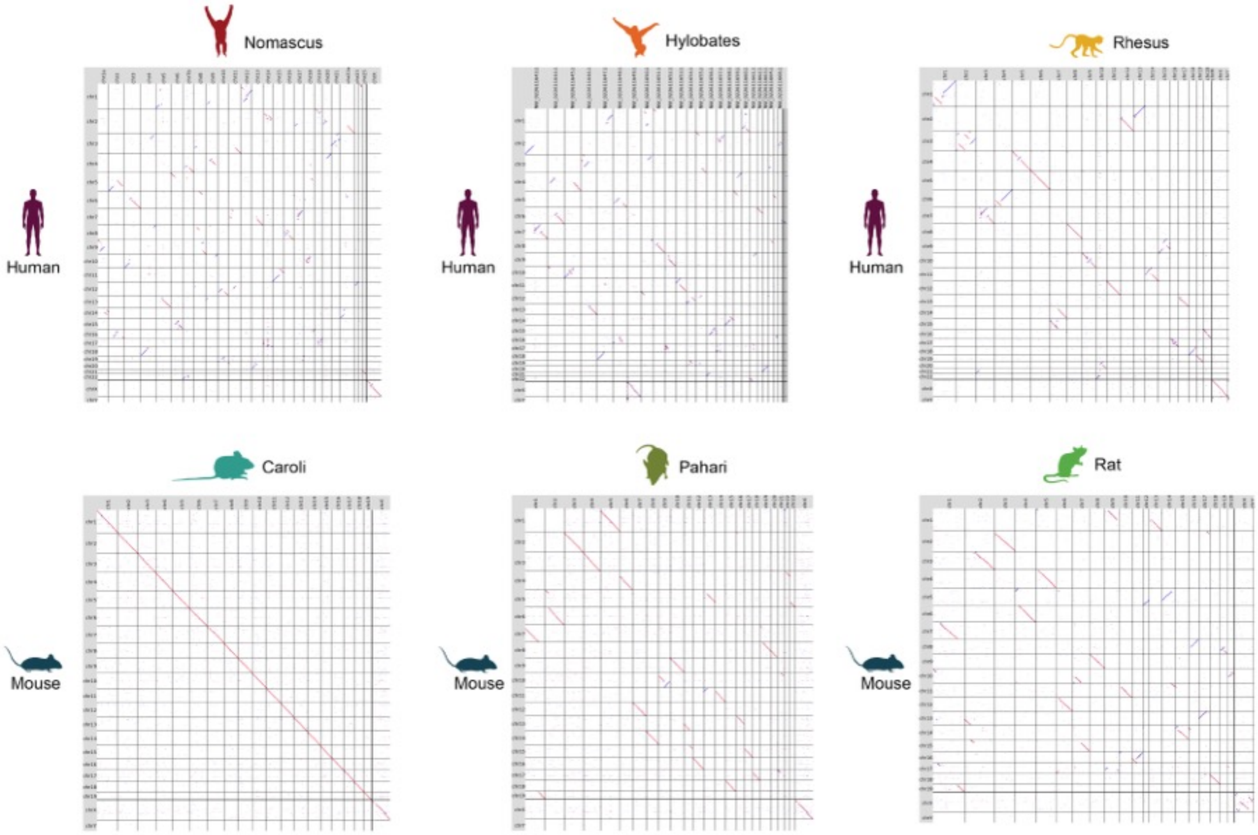

Supplementary Fig. 7: Identification of breaks of synteny (BOS) between species. Dot plots show pairwise comparisons between species genomes. With the exception of Caroli vs. mouse, all species show numerous breaks of synteny.

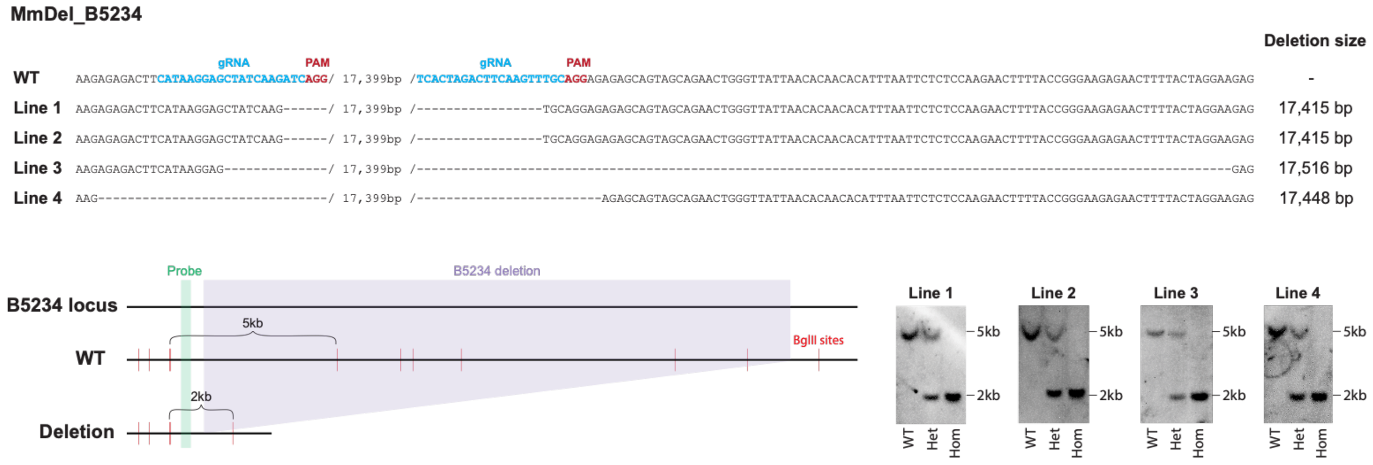

Supplementary Fig. 8 – Southern Blot and Sanger Sequencing Validation of CRISPR/*Cas9* deletions. Deletions for B5234 were confirmed by Sanger sequencing and Southern blot. Position of the probes is indicated.

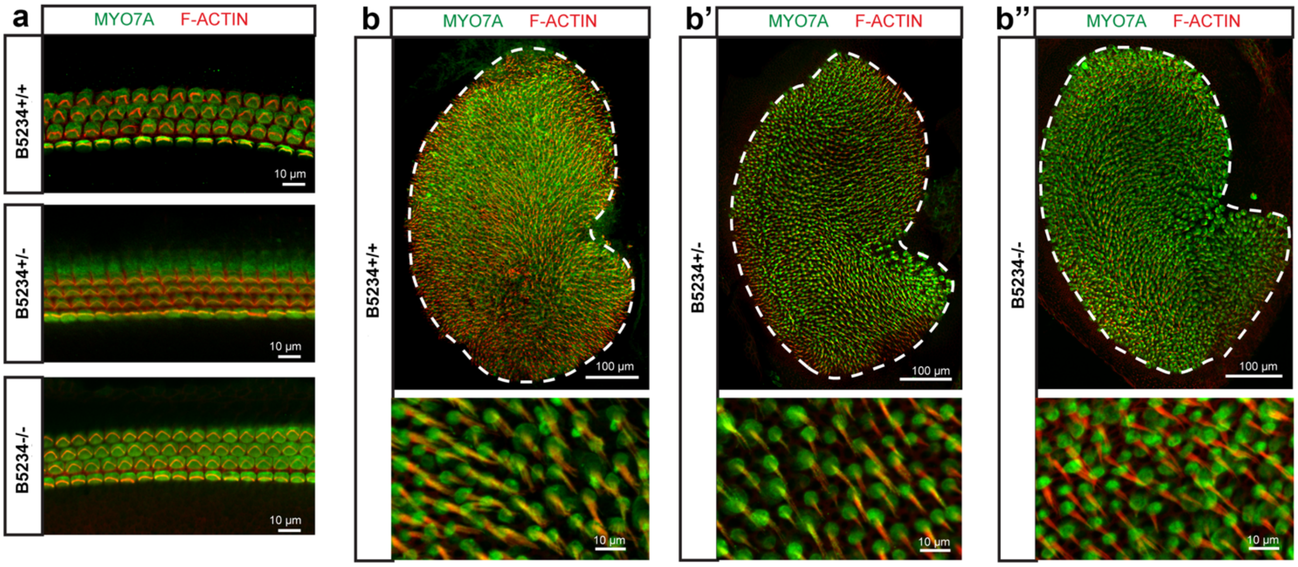

Supplementary Fig. 9 – Cochlear and utricular whole mount preparations show normal morphology. **a,** Whole mount preparations of the mouse cochlea in control, heterozygous, and homozygous genotypes show normal hair cells as marked by anti-myosin7a antibody (green) and normal hair bundles and orientation as detected by anti-F-actin (red). There are three rows of outer hair cells and one row of inner hair cells. **b-b",** Whole mount preparation of P3-P4 utricles demonstrate normal morphology and orientation of hair cells (anti-myosin7a, green) and hair bundles (anti-F-actin, red). The hair bundles are oriented appropriately along the line of polarity reversal. Wild-type n=2, heterozygous KO n = 3 and homozygous KO n = 3.

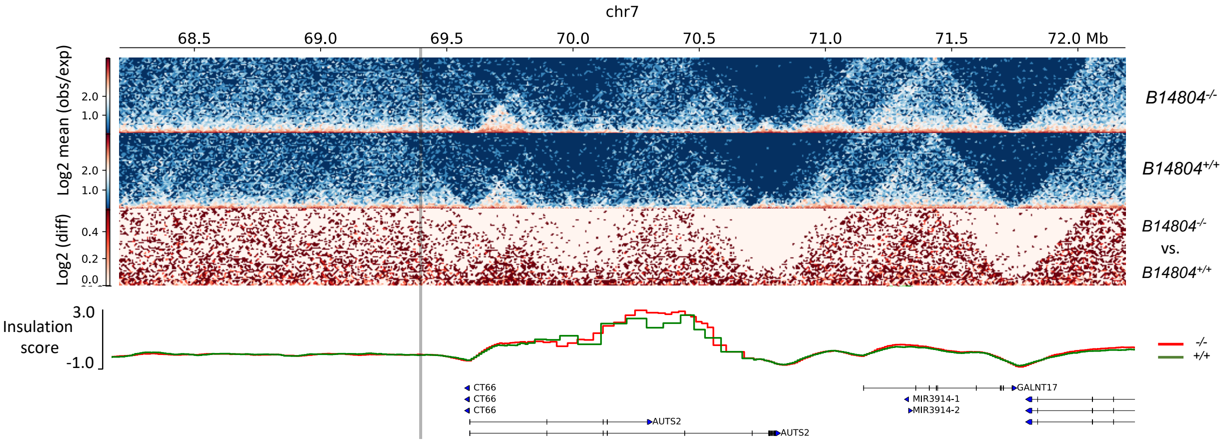

Supplementary Fig. 10 – Hi-C matrix of mutant and wild-type neurons near the *AUTS2* locus. Position of deletion is marked with gray vertical line.

Supplementary Note 1 - Sequence and assembly of *Nomascus leucogenys*

We generated an improved assembly of the *Nomascus leucogenys* using Pacbio continuous long-read sequencing (CLR) data. We generated ~92.5-fold sequence coverage of the previous female reference sample (Asia_NLE), assembled a squashed assembly using Falcon, and made the assembly publicly available (available on 7/7/2017 git hash 647e3726 and FALCON-integrate git hash 53444482, accession GCA_006542625). We performed an INDEL correction as previously described with freebayes v1.0.2-6-g3ce827d[1,2]. Bionano genomic data and BAC clone end-sequence data were used to scaffold assembly contigs. Finally, scaffolds were mapped to the previous gibbon reference (nomLeu3; UCSC version) and grouped into chromosomes. The overall assembled genome was estimated to be 2.84 Gb with approximately 510 gaps (Contig N50 12.78 Mbp) (**Table 1**).

	Asia_NLE_V1 (GCA_006542625.1)
Total sequence Length (Gbp)	2.844
Total ungapped length (Gbp)	2.829
Sequencing technology	PacBio RSII, Illumina, BioNano
Assembly method	FALCON
Gaps	510
Number of scaffolds	2,377
Scaffold N50 (bp)	35,886,894
Scaffold L50	29
Number of contigs	2,761
Contig N50 (bp)	12,782,853
Contig L50	72

Table 1 – Long-read *Nomascus leucogenys* genome assembly.

Supplementary Note 2 - Evolutionary classification of TAD boundaries

In order to perform cross-species comparisons and determine TAD boundary evolutionary conservation, we generated a union of all TAD boundaries from all eight species. To do this, TAD boundaries from each species were lifted to the human genome (hg38) coordinates using the UCSC LiftOver tool[3]. Boundaries from rat, Caroli, and Pahari were first lifted to the mouse genome (mm10) coordinates and then to the human genome, in order to improve lift over efficiency. Boundaries that failed to lift over, or those that lifted over to regions shorter than 5Kb, were not included in the union map (**Supplementary Data 3**). Next, we used bedtools[4] to merge all lifted-over boundaries located within 10Kb of each other in the human genome coordinates. To minimize the chances that a union boundary spanned several TAD units and overlapped with more than one TAD boundary from a single

species, we removed any union boundary that was longer than 190Kb following the merging. This length threshold corresponds to the shortest first quartile of TAD sizes across all eight species. Union TAD boundaries were then intersected with the lifted-over boundaries from each of the species to determine presence/absence of boundaries across species (**Supplementary Data 4**). Due to difficulties in obtaining liver tissue from apes, our cross-species Hi-C data originated from two different tissues (liver from rodents and rhesus, and LCL from apes). While this will likely result in some inaccuracies in estimating conservation of boundaries, we used patterns of TAD boundary distribution to validate that evolutionary relationship between species had a stronger effect on TAD organization compared to tissue of origin. We summarize patterns of cross-species boundary overlap in Upset plots[5] and found that boundaries from rhesus liver have more overlaps with boundaries from ape LCL, compared to rodent liver (**Supplementary Fig. 3**).
Moreover, logistic PCA analysis of TAD boundary presence/absence patterns (**Supplementary Fig. 1c**) shows grouping of samples based on phylogenetic order (primate vs. rodent) along the first principal component (PC1), indicating that phylogenetic relations explain a larger portion of the variation in TAD boundary organization compared to any other variable, including tissue source. Next, we used paradigms of boundary presence/absence across species to identify union boundaries belonging to each of the following evolutionary groups: 1) ultraconserved, i.e. boundaries shared among all eight species; 2) order-conserved (primate- or rodent-conserved), i.e. boundaries only present in all four species from each of the primate or rodent clades; 3) species-specific (human- or mouse-specific), i.e. boundaries found only in the species of human or mouse. Since our union boundary map does not include any non-human great-ape data, we used publicly available Hi-C data from chimpanzee and gorilla[6] (analyzed as described in the **Methods**) to remove any presumed human-specific boundaries shared with either of these two great-ape species. It should be noted that since our approach relies on successful lift over of sequences, cross-species variation in efficiency of lift over can be misinterpreted as lack of boundary conservation and result in an underestimation of conserved boundaries.

Supplementary Note 3 - <u>Permutation analyses</u>

Permutation tests were performed to compare the observed insulation scores for each boundary conservation group to random expectation. Briefly, we randomly selected 100 TAD boundaries in the human and mouse genomes, with the proportion of boundaries overlapping genes matching that of the TAD boundary evolutionary class being tested. We repeated this process 1000 times, and the portion of times out of 1000 when the median insulation scores in the random set of boundaries were more extreme than the observed median for the conservation group, was the empirical p-value (**Supplementary Fig. 6**).

To investigate association of human-specific TAD boundary deletion with human disease, we used a comprehensive list of publicly available copy number variants (CNVs) identified from 29,085 children with developmental delay (i.e. “case”) and 19,584 healthy controls (i.e. “control”)[7]. We only considered CNVs identified on the assembled hg38 chromosomes, resulting in a total of 29,110 CNVs in the case dataset and 273,663 in the control. We used an approach similar to Fudenberg et al.[8] to compare the rate of recurrent human-specific TAD boundary deletions in cases and controls. Briefly, we used bedtools shuffle[4] to permute the position of case and control CNVs, while restricting the shuffled position to their original chromosome. We repeated this process 100 times and each time calculated the number of permutated CNVs covering each human-specific TAD boundary. For each boundary we selected the 95th percentile of the permutated CNV boundary coverage as a threshold to identify human-specific boundaries that are recurrently deleted in case subjects (335 boundaries) and controls. We then used GraphPad (www.graphpad.com) to perform a two-tailed Fisher’s exact test and compare the total number of significantly recurrent deleted human-specific boundaries vs. those that are non-recurrent in case and control CNV datasets (2x2 contingency table).

Supplementary Note 4 - Histone and CTCF ChIP-seq

We used a combination of public and newly generated H3K4me1, H3K27ac, H3K4me3, H3K27me3, and CTCF ChIP-seq data from one female and one male from each of the eight species examined in this study (**Supplementary Data 2**). All rodent and rhesus ChIP-seq libraries were generated from liver tissue, while LCL was used for the rest of the species. For liver samples, 50-100mg of lightly chopped tissue was fixed with formaldehyde before quenching with glycine and then needle homogenized. The tissue slurry was pelleted and then lysed with 100ul of Lysis Buffer per 10mg of tissue. Lysates then followed the same ChIP-seq protocol as the LCL pellets, which has been described before[32,55]. Antibodies were used in the following amounts for all samples: 1.5ul H3K4me1 (ab8895, Abcam), 1ul H3K4me3 (ab8580, Abcam), 1.5ul H3K27ac (ab4729, Abcam), 2ul H3K27me3 (39155, Active Motif) and 10ul CTCF (3418s, Cell Signaling Technologies). All ChIP-seq libraries were prepared using the NEBNext Ultra II DNA Library Prep Kit for Illumina (New England Biolabs) without size selection, and sequenced on the Illumina HiSeq2500 or NovaSeq6000. To minimize technical variation across datasets, we only used R1 reads if ChIP-seq datasets were sequenced paired-end. After QC, reads were aligned to their respective genome assemblies using bowtie2[56], with default single-end settings. Reads with mapping quality <30 were removed. Using the ChromHMM program[9], we determined eight chromatin states based on the combination of epigenetic marks and calculated the enrichment of each state at TAD boundaries in each evolutionary group. We also used MACS2[10] (--nomodel --extsize 300) to identify significant CTCF ChIP-seq peaks within each species. We then combined CTCF peaks

between replicates, and merged peaks within 200bp using bedtools[4]. The merged CTCF peaks were intersected with TAD boundary coordinates to identify CTCF binding sites within TAD boundaries in each species. We also merged all histone and CTCF ChIP-seq alignments from the female and male replicates and used deeptools[11] to visualize the collective intensity of each epigenetic mark in RPKM units (reads per kilobase, per million mapped reads) at and around TAD boundaries in each species. Lastly, we used MACS2[58] to generate UCSC genome browser tracks of log2 fold-enrichment (against input) for CTCF ChIP-seq data.

To compare the chromatin structure of evolutionary TAD groups, we used chromatin state annotation described above to calculate “fold enrichment” of each state at evolutionary TAD boundary groups using (C/A)/(B/D), where: “A” is the genome-wide number of bases in the state, “B” is the collective length (bp) of the specific evolutionary group of TAD boundaries in the genome, “C” is the collective length (bp) of overlaps between chromatin state and TAD boundary group, and “D” is the total size of genome (in bp).

Supplementary Note 5 - Generation of *B5234$^{-/-}$* knockout mice

The *B5234$^{-/-}$* line was generated using two gRNAs designed with the gRNA design tool on the Integrated DNA Technologies (IDT) website (**Supplementary Data 11**). These gRNAs were selected based on low off-target and high on-target scores. The TAD deletion was generated using i-GONAD[12]. Briefly, crRNA (IDT) and tracrRNA (IDT) were mixed together (final concentration 100 μM each) and incubated at 92 °C for 2 minutes, then left at room temperature for 10 minutes to prepare the crRNA/tracrRNA complex. The genome-editing mixture (30 μM crRNA/tracrRNA complex, 1 mg/ml Cas9 protein (IDT), Opti-MEM) was incubated at 37 °C for 10 minutes. Estrus female FVB mice (Jackson Laboratory, catalog no. 001800) were mated to male mice the night before. The presence of copulation plugs was confirmed by visual inspection the next morning, and the females having plugs were designated as Day 0.5 of gestation at noon and Day 0.7 of gestation at 16:00. Females on Day 0.7 were used for oviduct electroporation. Mice were anesthetized using isoflurane, and 1-2μl of the genome-editing mixture was injected into the oviduct lumen upstream of the ampulla. Immediately following injection, the oviduct was covered with a piece of wet paper soaked in phosphate buffered saline (PBS) and then grasped by tweezer-type electrodes (Bulldog Bio). The electroporation was performed using a square-wave pulse generator BTXECM830 (BTX Genetronics Inc.) with 8 pulses of 50 V at 5 msec wavelength. After electroporation, the oviducts were placed in their original position and the muscle layer incision was sutured using absorbable suture chromic gut. The coat layer incision was closed by AutoClip kit (Fine Science Tools, catalog no. 12022-09). The animals were kept on a 37 °C warming pad during surgery and monitored for anesthesia recovery following surgery.

In order to validate deletions in surviving offspring, genomic DNA was extracted from mouse tail clippings and PCR amplified using the KAPA Mouse Genotyping Kit (Roche) following the manufacturer's protocol. PCR amplicons were visualized on agarose gel to determine genotype. The primers and expected band sizes for each genotype can be found in **Supplementary Data 11**. For further validation, we also performed Southern blot on genomic DNA extracted from mouse tail clippings. Genomic DNA was digested using BglII (New England Biolabs). Digestions were run on agarose gel and transferred onto nylon membranes via capillary transfer. Digoxigenin (DIG)-labeled DNA probes were designed (chr2:115,839,949-115,840,248) and were amplified using the PCR DIG Probe Synthesis Kit (Sigma-Aldrich) before being hybridized to the membranes. The hybridized probe was detected with anti-digoxigenin Fab fragments (Sigma-Aldrich) and CDP-star (Sigma-Aldrich) chemiluminescence was visualized and imaged using the FluorChem E (ProteinSimple). Breakpoints of the deletions were also confirmed using Sanger sequencing (**Supplementary Fig. 8**).

Supplementary Note 6 - Phenotyping *B5234*$^{-/-}$ mice

ChIP-seq, qRT-PCR and Capture Hi-C on heart

We used freshly frozen hearts from five-day old B5234$^{-/-}$ and B5234$^{+/+}$ mice to generate CTCF ChIP-seq libraries as described above (n= 3, per genotype). Raw CTCF ChIP-seq data was aligned to the mm10 reference genome using bowtie2[13], with default settings. Reads with mapping quality <30 were removed. We used MACS258 (--nomodel --extsize 300) to generate fold-enrichment tracks to confirm removal of the target CTCF site following deletion.

Total RNA was extracted from hearts of 5 day old (P5) B5234$^{-/-}$ mice using TRIzol reagent (Invitrogen) and converted to cDNA using ReverTra Ace qPCR-RT master mix with genomic DNA (gDNA) remover (Toyobo, FSQ-301) following the manufacturer's protocol. qRT-PCR was performed using SsoFast EvaGreen Supermix (Bio-Rad) on QuantStudio 6 Real Time PCR system. Statistical analysis was performed using ddct method and GAPDH as control with two-tailed unpaired t-test. Gene expression results were generated using mean values for n=3 biological replicates. Primer sequences used for qPCR are reported in **Supplementary Data 11**.

Per genotype, we also generated two pools each comprised of two freshly frozen hearts from five-day old mice to generate Capture Hi-C libraries. Capture Hi-C libraries were generated by using the Agilent SureSelect Target Enrichment Kit. Briefly, Hi-C libraries were prepared as described in the **Methods** and then hybridized with custom probes before enrichment via Dynabeads MyOne Streptavidin Beads T1 (ThermoFisher). After library enrichment, a post-capture PCR was conducted using 14 cycles and libraries were paired-end sequenced at OHSU Massively Parallel Sequencing Shared Resource (MPSSR). Raw sequencing data from mutant and control tissue was processed similar to Hi-C libraries

as described in the **Methods**, with minor differences. Briefly, raw Capture Hi-C data was aligned to *mm10* genome using HiCUP[14]. Pairtools (https://github.com/mirnylab/pairtools) was used to further process data and merge interaction matrices across replicates in each genotype. We used HiCExplorer[15] to obtain insulation scores along the targeted loci.

Heart histology and image analysis

To characterize the histology and morphology of *B5234*$^{-/-}$ hearts, we harvested fresh hearts from three-month old *B5234*$^{-/-}$ mice and *B5234*$^{+/+}$ mice (n=4/group) and fixed them in 4% paraformaldehyde for over 24 hours. We then embedded the fixed tissue in paraffin and sectioned them at 4μm intervals. After deparaffinization, slides were stained with hematoxylin and eosin (H&E) via standard methods. First, visual inspection of the H&E sections for each heart was performed to determine morphological characteristics and whether the hearts were malformed. To quantify differences, the thickness of the left ventricle (LV) and right ventricle (RV) walls from approximately corresponding sections displaying atrio-ventricular valves (for consistency) were measured. Three wall thickness measurements were taken from each heart and ventricle wall (LV and RV). To further quantify LV wall compaction, images from corresponding regions of interest within the LV wall were extracted and processed using Dragonfly (Object Research System) software using thresholding to segment extracellular space within the LV wall. The percentage of the surface segmented, that is the percentage of the region of interest surface occupied by extracellular space from 3 approximately corresponding regions in each heart, was then quantified.

Auditory brainstem response (ABR) test

Mouse hearing was tested in seven-month old mice for all three genotypes by measuring auditory brainstem response (ABR) thresholds in response to broadband clicks and 8, 16, and 32-kHz pure-tone pips in the sound field using a standard commercial system (RZ6, Tucker-Davis Technologies) in a soundproof chamber[16]. Thresholds across genotypes were compared using one-way ANOVA with Bonferroni correction for multiple comparisons.

Utricular and cochlear histology

Temporal bones were isolated from P3-P4 animals of all three genotypes following animal sacrifice (wild-type n=2, heterozygous KO= 3, and homozygous KO= 3). Isolated temporal bones were fixed using 4% paraformaldehyde on ice followed by PBS washes. Utricles and cochleae were microdissected in cold PBS. Wholemount utricles and cochleae were prepared by mounting to coverslips covered with 1uL CellTak adhesive in PBS. Utricles were incubated in EDTA for 40 minutes

at room temperature to dissolve the otoconia. Tissues were washed with 0.1% Triton X-100 in PBS three times for 20 minutes each. Antigen blocking was performed using 5% donkey serum, 0.1% Triton X-100, 1% bovine serum albumin (BSA, Thermo Fisher Scientific, BP1600-100), and 0.02% sodium azide (NaN_3) in PBS at pH 7.4 for 1-2 hours at room temperature. Samples were then incubated with the primary antibody overnight at 4°C. Specimens were washed the next day with 0.1% Triton X-100 in PBS three times at room temperature for 5 minutes each and incubated with secondary antibodies with DAPI for 2 hours at room temperature. Tissues were then washed with PBS for 5 minutes each and mounted using Pro-Long Gold mounting media. The primary antibodies used included rabbit anti-myosin7a (1:1000, Proteus Biosciences) and fluorescent conjugated anti-F-actin (1:100, Sigma). Secondary antibodies included Alexa 488 (1:400, Invitrogen), Alexa 546 (1:400, Invitrogen), or Alexa 647 (1:400, Invitrogen). Images were acquired using a Zeiss Apotome 3 M2 AxioImager or Nikon A1R confocal. CZI or ND files were opened in Fiji (NIH Image J) for intensity adjustment and processed in Adobe Photoshop and Illustrator for final figure preparation (**Supplementary Fig. 9**).

References for supplementary information

1. Kronenberg, Z.N. *et al.* High-resolution comparative analysis of great ape genomes. *Science* **360**(2018).
2. Mao, Y. *et al.* A high-quality bonobo genome refines the analysis of hominid evolution. *Nature* **594**, 77-81 (2021).
3. Kuhn, R.M., Haussler, D. & Kent, W.J. The UCSC genome browser and associated tools. *Brief Bioinform* **14**, 144-61 (2013).
4. Quinlan, A.R. & Hall, I.M. BEDTools: a flexible suite of utilities for comparing genomic features. *Bioinformatics* **26**, 841-2 (2010).
5. Lex, A., Gehlenborg, N., Strobelt, H., Vuillemot, R. & Pfister, H. UpSet: Visualization of Intersecting Sets. *IEEE Trans Vis Comput Graph* **20**, 1983-92 (2014).
6. Yang, Y., Zhang, Y., Ren, B., Dixon, J.R. & Ma, J. Comparing 3D Genome Organization in Multiple Species Using Phylo-HMRF. *Cell Syst* **8**, 494-505 e14 (2019).
7. Coe, B.P. *et al.* Refining analyses of copy number variation identifies specific genes associated with developmental delay. *Nat Genet* **46**, 1063-71 (2014).
8. Fudenberg, G. & Pollard, K.S. Chromatin features constrain structural variation across evolutionary timescales. *Proc Natl Acad Sci U S A* **116**, 2175-2180 (2019).
9. Ernst, J. & Kellis, M. ChromHMM: automating chromatin-state discovery and characterization. *Nat Methods* **9**, 215-6 (2012).
10. Liu, T. Use model-based Analysis of ChIP-Seq (MACS) to analyze short reads generated by sequencing protein-DNA interactions in embryonic stem cells. *Methods Mol Biol* **1150**, 81-95 (2014).
11. Ramirez, F., Dundar, F., Diehl, S., Gruning, B.A. & Manke, T. deepTools: a flexible platform for exploring deep-sequencing data. *Nucleic Acids Res* **42**, W187-91 (2014).
12. Gurumurthy, C.B. *et al.* Creation of CRISPR-based germline-genome-engineered mice without ex vivo handling of zygotes by i-GONAD. *Nat Protoc* **14**, 2452-2482 (2019).
13. Langmead, B. & Salzberg, S.L. Fast gapped-read alignment with Bowtie 2. *Nat Methods* **9**, 357-9 (2012).
14. Wingett, S. *et al.* HiCUP: pipeline for mapping and processing Hi-C data. *F1000Res* **4**, 1310 (2015).

15. Wolff, J. *et al.* Galaxy HiCExplorer 3: a web server for reproducible Hi-C, capture Hi-C and single-cell Hi-C data analysis, quality control and visualization. *Nucleic Acids Res* **48**, W177-W184 (2020).
16. Li, J. *et al.* Deletion of Tmtc4 activates the unfolded protein response and causes postnatal hearing loss. *J Clin Invest* **128**, 5150-5162 (2018).
