# Supplementary material for: TAD evolutionary and functional characterization reveals diversity in mammalian TAD boundary properties and function
Source: Nat Commun. 2023 Dec 7;14:8111. doi: 10.1038/s41467-023-43841-8 (PMC10703881; doi:10.1038/s41467-023-43841-8)
Supplement: Supplementary file 3 — Description of Additional Supplementary Files [file 41467_2023_43841_MOESM3_ESM.pdf]

Description of Additional Supplementary Files

File name: Supplementary Data 1

Description: Hi-C sequencing summary statistics and number of TADs and boundaries for each species.

File name: Supplementary Data 2

Description: Chip-seq summary statistics

File name: Supplementary Data 3

Description: TAD boundary LiftOver rates for each species.

File name: Supplementary Data 4

Description: Union boundaries and classification in evolutionary groups

File name: Supplementary Data 5

Description: Classification of boundaries based on the number of species sharing them

File name: Supplementary Data 6

Description: Number of synteny blocks and synteny breakpoints between target and query genomes

File name: Supplementary Data 7

Description: Odds ratio test results from examining overlap of BOS and TAD boundaries across species.

File name: Supplementary Data 8

Description: List of breaks of synteny

File name: Supplementary Data 9

Description: GREAT GO term analysis on Ultra-conserved boundaries.

File name: Supplementary Data 10

Description: GREAT GO term analysis on human-specific boundaries.

File name: Supplementary Data 11

Description: Primers and gRNA used in this study.
