# Supplementary material for: TAD evolutionary and functional characterization reveals diversity in mammalian TAD boundary properties and function
Source: Nat Commun. 2023 Dec 7;14:8111. doi: 10.1038/s41467-023-43841-8 (PMC10703881; doi:10.1038/s41467-023-43841-8)
Supplement: Supplementary file 15 — Reporting Summary [file 41467_2023_43841_MOESM15_ESM.pdf]

nature portfolio

Corresponding author(s): Lucia Carbone

Last updated by author(s): 10/23/2023

Reporting Summary

Nature Portfolio wishes to improve the reproducibility of the work that we publish. This form provides structure for consistency and transparency in reporting. For further information on Nature Portfolio policies, see our Editorial Policies and the Editorial Policy Checklist.

Statistics

For all statistical analyses, confirm that the following items are present in the figure legend, table legend, main text, or Methods section.

n/a	Confirmed	
☐	☒	The exact sample size (*n*) for each experimental group/condition, given as a discrete number and unit of measurement
☐	☒	A statement on whether measurements were taken from distinct samples or whether the same sample was measured repeatedly
☐	☒	The statistical test(s) used AND whether they are one- or two-sided<br>*Only common tests should be described solely by name; describe more complex techniques in the Methods section.*
☐	☒	A description of all covariates tested
☐	☒	A description of any assumptions or corrections, such as tests of normality and adjustment for multiple comparisons
☐	☒	A full description of the statistical parameters including central tendency (e.g. means) or other basic estimates (e.g. regression coefficient) AND variation (e.g. standard deviation) or associated estimates of uncertainty (e.g. confidence intervals)
☐	☒	For null hypothesis testing, the test statistic (e.g. *F*, *t*, *r*) with confidence intervals, effect sizes, degrees of freedom and *P* value noted<br>*Give P values as exact values whenever suitable.*
☒	☐	For Bayesian analysis, information on the choice of priors and Markov chain Monte Carlo settings
☒	☐	For hierarchical and complex designs, identification of the appropriate level for tests and full reporting of outcomes
☐	☒	Estimates of effect sizes (e.g. Cohen's *d*, Pearson's *r*), indicating how they were calculated

Our web collection on statistics for biologists contains articles on many of the points above.

Software and code

Policy information about availability of computer code

Data collection	The software used to collect the images from mouse heart tissue was Dragonfly (Object Research System). The software used to collect the Utricles and cochleae images was Zen Blue Software (v3.3) on AxioImager M2 Apotome 3 Nikon A1R NIS Elements Software (v5.3). The software used to collect the ABR data is BioSigRZ (v5.7.0) (embedded into the RZ6 (Tucker-Davis Technologies, Alachua, FL) ABR system).
Data analysis	The following publicly available software were used to analyze data: HiCExplorer 3.7.2 (https://hicexplorer.readthedocs.io/en/latest/), HiCRes (https://github.com/ClaireMarchal/HiCRes), macs2 2.2.5 (https://github.com/macs3-project/MACS/wiki/Install-macs2), Bowtie2 2.3.2 (https://sourceforge.net/projects/bowtie-bio/), samtools 1.3.1 (https://sourceforge.net/projects/samtools/files/samtools/1.3.1/), ChromHMM 1.20 (http://compbio.mit.edu/ChromHMM/), TEanalysis tool, Bedtools v2.30.0 (https://bedtools.readthedocs.io/en/latest/), STAR 2.7.8a (https://github.com/alexdobin/STAR), MultiHiCcompare (3.17) (https://www.bioconductor.org/packages/release/bioc/html/multiHiCcompare.html).<br><br>The following custom scripts were also used and are available on github: LASTZ (https://github.com/carbonelab/lastz-pipeline), Pairwise genome alignments: (https://github.com/carbonelab/axtToSyn), Snakemake workflow for QC and processing of Hi-C data (https://github.com/carbonelab/hic_workflow).

For manuscripts utilizing custom algorithms or software that are central to the research but not yet described in published literature, software must be made available to editors and reviewers. We strongly encourage code deposition in a community repository (e.g. GitHub). See the Nature Portfolio guidelines for submitting code & software for further information.

Data

Policy information about availability of data

All manuscripts must include a data availability statement. This statement should provide the following information, where applicable:

- Accession codes, unique identifiers, or web links for publicly available datasets
- A description of any restrictions on data availability
- For clinical datasets or third party data, please ensure that the statement adheres to our policy

All Hi-C, ChIP-seq and Capture Hi-C data generated as part of this project study are available at the Gene Expression Omnibus (GEO) under the accession number: GSE197926 (https://www.ncbi.nlm.nih.gov/geo/query/acc.cgi?&acc=GSE197926). The reference genomes used in this study are: Human, hg38 (https://genome.ucsc.edu/cgi-bin/hgGateway?hgsid=1738767972_ryzhdYsyt1Nt9sG6pkyJwcl4cRAA) ; Nomascus leucogenys, Asia_NLE_v1 (https://www.ncbi.nlm.nih.gov/data-hub/genome/GCF_006542625.1/); Hylobates moloch, HMol_V3 (https://www.ncbi.nlm.nih.gov/datasets/genome/?taxon=81572); Rhesus macaque, rheMac10 (https://genome.ucsc.edu/cgi-bin/hgGateway?hgsid=1738767972_ryzhdYsyt1Nt9sG6pkyJwcl4cRAA); Mus musculus, mm10 (https://genome.ucsc.edu/cgi-bin/hgGateway?hgsid=1738767972_ryzhdYsyt1Nt9sG6pkyJwcl4cRAA); Mus pahari, PAHARI_EIJ_v1.1 (https://www.ncbi.nlm.nih.gov/datasets/genome/GCF_900095145.1/); Mus caroli, CAROLI_EIJ_v1.1 (https://www.ncbi.nlm.nih.gov/datasets/genome/GCF_900094665.1/); Rattus norvegicus, rn6 (https://genome.ucsc.edu/cgi-bin/hgGateway?hgsid=1738767972_ryzhdYsyt1Nt9sG6pkyJwcl4cRAA). Public Hi-C datasets used in this study were obtained from GEO under the following accession numbers: GSE128800 (https://www.ncbi.nlm.nih.gov/geo/query/acc.cgi?acc=GSE128800), SRR6675327 (https://www.ncbi.nlm.nih.gov/sra?term=SRR6675327), GSM3682186 (https://www.ncbi.nlm.nih.gov/geo/query/acc.cgi?acc=GSM3682186) and GSM3682187 (https://www.ncbi.nlm.nih.gov/geo/query/acc.cgi?acc=GSM3682187). Public ChIP-seq data was accessed at GEO under the following accession numbers GSE50893 (https://www.ncbi.nlm.nih.gov/geo/query/acc.cgi?acc=GSE50893), GSE136963 (https://www.ncbi.nlm.nih.gov/geo/query/acc.cgi?acc=GSE136963) , GSE136968 (https://www.ncbi.nlm.nih.gov/geo/query/acc.cgi?acc=GSE136968), GSE60269 (https://www.ncbi.nlm.nih.gov/geo/query/acc.cgi?acc=GSE60269), GSM1087083 (https://www.ncbi.nlm.nih.gov/geo/query/acc.cgi?acc=GSM1087083) and at the EMBL-EBI under https://www.ebi.ac.uk/biostudies/arrayexpress/studies/E-MTAB-1511/sdrf.

Research involving human participants, their data, or biological material

Policy information about studies with human participants or human data. See also policy information about sex, gender (identity/presentation), and sexual orientation and race, ethnicity and racism.

Reporting on sex and gender	N/A
Reporting on race, ethnicity, or other socially relevant groupings	N/A
Population characteristics	N/A
Recruitment	N/A
Ethics oversight	N/A

Note that full information on the approval of the study protocol must also be provided in the manuscript.

Field-specific reporting

Please select the one below that is the best fit for your research. If you are not sure, read the appropriate sections before making your selection.

☐ Life sciences ☐ Behavioural & social sciences ☒ Ecological, evolutionary & environmental sciences

For a reference copy of the document with all sections, see nature.com/documents/nr-reporting-summary-flat.pdf

Ecological, evolutionary & environmental sciences study design

All studies must disclose on these points even when the disclosure is negative.

Study description	Hi-C and ChIP-seq data was generated and compared for 8 species, 4 primates and 4 rodents (2 biological replicates per species), in order to identify shared and species-specific 3D genomics features, with a focus on boundaries of Topologically Associating Domains (TADs). Capture Hi-C data, ChIP-seq, qPCR and imaging data were obtained on heart tissue from a mouse model carrying a CRISPR deletion (B5234-/-) and wild type (B5234+/+) for comparison (N=4). Imaging data for the same mouse model and wild-type were obtained on utricles and cochleae. Finally, Hi-C and qPCR data were obtained on WCT11-Ngn2 cells after they were underwent a CRISPR deletion and were differentiated to glutamatergic neurons (N=2 cell lines).
Research sample	The species involved in the study are: Human (order: Primates; family: Hominidae; genus: Homo; species: Homo sapiens); Nomascus leucogenys (order: Primates; family: Hylobatidae; genus: Nomascus; species: Nomascus leucogenys); Hylobates moloch (order: Primates; family: Hylobatidae; genus: Hylobates; species: Hylobates moloch); Rhesus macaque (order: Primates; family: Cercopithecidae; genus: macaque; species: Macaca mulatta); Mouse (order: Rodentia; family: Muridae; genus: Mus; species: Mus musculus); Mus pahari (order: Rodentia; family: Muridae; genus: Mus; species: M. pahari); Mus caroli (order: Rodentia; family:

Muridae; genus: Mus; species: M. caroli); Rat (order: Rodentia; family: Muridae; genus: Rattus; Rattus norvegicus). For each species we used one male and one female. Ages are unknown. The samples do not represent any specific population.

Mus musculus (FVB/NJ (Jackson Lab; 001800) carried a CRISPR/deletion.

Sampling strategy

No power analysis was performed. Instead, we selected two individuals for each species, one male and one female, in order to have two biological replicates and merge the data if the replicates had high correlation.

Data collection

All Hi-C libraries, with the exception of the human and the great apes, were generated in the Carbone Lab by Kimberly Nevonen, Samantha Ward, Jarod Herrera. We used liver tissue and lymphoblastoid cell lines (LCLs) to generate Hi-C libraries with the Arima Hi-C Kit following manufacturer's protocol. Briefly, ~5ug frozen pellets of the homogenized fixed liver (for mus musculus, mus pahari, mus caroli, Rattus norvegicus and Rhesus macaque) or LCL (for human, Nomascus leucogenys, Hylobates moloch) were lysed and conditioned before chromatin digestion. The digested chromatin was then filled in and biotinylated before ligation. Next, chromatin was protein-digested and reverse crosslinking overnight, followed by purification. The purified DNA was then sonicated using the bioruptor pico (Diagenode) and size selected before library preparation using the NEB DNA Ultra II, following Arima's protocol. The Hi-C DNA was bound to streptavidin beads before enzymatic end prep, adaptor ligation, DNA release by heat incubation, and lastly, PCR to barcode and amplify the libraries. Libraries were sequenced on the Illumina HiSeq2500 or NovaSeq6000.

Capture Hi-C was used to analyze mice hearts after CRISPR deletion and was performed by Cora E. Layman. Two freshly frozen hearts from 5 day old mice from each genotype (B5234-/- and B5234+/+) were pooled. Capture Hi-C libraries were generated by using the Agilent SureSelect Target Enrichment Kit. Briefly, Hi-C libraries were prepared as described above and then hybridized with custom probes before enrichment via Dynabeads MyOne Streptavidin Beads T1 (ThermoFisher). After library enrichment, a post capture PCR was conducted using 14 cycles and libraries were paired-end sequenced. Libraries were sequenced on the NovaSeq6000.

ChIP-seq libraries were generated by Kimberly Nevonen and Jarod Herrera. We used a combination of public and newly generated H3K4me1, H3K27ac, K3K4me3, H3K27me3 and CTCF ChIP-seq data from one female and one male from each of the eight species examined in this study (Supplementary Table 2). All rodent and rhesus ChIP-seq libraries were generated from liver tissue, while LCL was used for the rest of the species. For liver samples, 50-100 mg of lightly chopped tissue was fixed with formaldehyde before quenching with glycine and then needle homogenized. The tissue slurry was pelleted and then lysed with 100ul of Lysis Buffer per 10mg of tissue. Lysates then followed the same ChIP-seq protocol as the LCL pellets, which has been described before32,55. Antibodies were used in the following amounts for all samples: 1.5ul H3K4me1 (ab8895, Abcam), 1ul H3K4me3 (ab8580, Abcam), 1.5ul H3K27ac (ab4729, Abcam), 2ul H3K27me3 (39155, Active Motif) and 10ul CTCF (3418s, Cell Signaling Technologies). All ChIP-seq libraries were prepared using the NEBNext Ultra II DNA Library Prep Kit for Illumina (New England Biolabs) without size selection, and sequenced on the Illumina HiSeq2500 or NovaSeq6000.

Imaging data from the mice heart tissues were collected by the Histology and Light Microscopy Core Facility, Gladstone Institutes, (San Francisco). To characterize the histology and morphology of mutant (B5234-/-) hearts, fresh hearts were harvested from B5234-/- three months old mice and B5234+/+ mice (n=4/group) and fixed in 4% paraformaldehyde for over 24 hours. The fixed tissue was then embedded in paraffin and sectioned them at 4µm intervals. After deparaffinization, slides were stained with hematoxylin and eosin (H&E) via standard methods. Each heart was first visually inspected to determine morphological characteristics and malformations. Then the thickness of the left (LV) and right ventricle (RV) walls were measured from approximately corresponding sections displaying atrio-ventricular valves (for consistency). Three wall thickness measurements were taken from each heart and ventricle wall (LV and RV). To further quantify LV wall compaction, images were extracted from corresponding regions of interest within the LV wall. Those images were processed using Dragonfly (Object Research System) software using thresholding to segment extracellular space within the LV wall. The percentage of the surface segmented, that is the percentage of the region of interest surface occupied by extracellular space from 3 approximately corresponding regions in each heart, was then quantified.

qPCR experiments on the heart tissues from B5234-/- and B5234+/+ mice were performed by Weiyu Li and Rory R. Sheng at UCSF. Total RNA was extracted from hearts of P5 B5234-/- mice using TRIzol reagent (Invitrogen) and converted to cDNA using ReverTra Ace qPCR-RT master mix with genomic DNA (gDNA) remover (Toyobo, FSQ-301) following the manufacturer's protocol. qRT-PCR was performed using SsoFast EvaGreen supermix (Bio-Rad) on QuantStudio 6 Real Time PCR system. Statistical analysis was performed using ddct method and GAPDH as control with two-tailed unpaired t-test. Gene expression results were generated using mean values for n=3 biological replicates. Primer sequences used for qPCR are reported in Supplementary Table 11.

Mouse hearing tests were performed by Ian R. Matthews and Dylan. K. Chan at UCSF. Mouse hearing was tested in seven-month-old mice by measuring auditory brainstem response (ABR) thresholds in response to broadband clicks and 8, 16, and 32-kHz pure-tone pips, in the sound field using a standard commercial system (RZ6, Tucker-Davis Technologies) in a soundproof chamber12. Thresholds across genotypes were compared using one-way ANOVA with Bonferroni correction for multiple comparisons.

Imaging data from the mice ear were collected by Sarah A. Easow and Taha A. Jan at UCSF. Temporal bones were isolated from P3-P4 animals of all three genotypes following animal sacrifice. Isolated temporal bones were fixed using 4% paraformaldehyde on ice followed by Phosphate Buffered Saline (PBS) washes. Utricles and cochlea were microdissected in cold PBS. Wholemount utricles and cochlea were prepared by mounting to coverslips covered with 1uL CellTak adhesive in PBS. Utricles were incubated in EDTA for 40 minutes at room temperature to dissolve the otoconia. Tissues were washed with 0.1% Triton X-100 in PBS three times for 20 minutes each. Antigen blocking was performed using 5% donkey serum, 0.1% Triton X-100, 1% bovine serum albumin (BSA, Thermo Fisher Scientific, BP1600-100), and 0.02% sodium azide (NaN3) in PBS at pH 7.4 for 1-2 hours at room temperature. Samples were then incubated with the primary antibody overnight at 4°C. Specimens were washed the next day with 0.1% Triton X-100 in PBS three times at room temperature for 5 minutes each and incubated with secondary antibodies with DAPI for 2 hours at room temperature. Tissues were then washed with PBS for 5 minutes each and mounted using Pro-Long Gold mounting media. The primary antibodies used included rabbit anti-myosin7a (1:1000, Proteus Biosciences) and fluorescent conjugated anti-F-actin (1:100, Invitrogen). Secondary antibodies included Alexa 488 (1:400, Invitrogen), Alexa 546 (1:400, Invitrogen), or Alexa 647 (1:400, Invitrogen). Images were acquired using a Zeiss Apotome 3 M2 Axioimager or Nikon A1R confocal. CZI or ND files were opened in Fiji (NIH Image J) for intensity adjustment and processed in Adobe Photoshop and Illustrator for final figure preparation.

The CRISPR deletion on the WTC11-Ngn2 cells was performed by Lana Harshman at UCSF. CRISPR knockout assays targeted an 11Kb region (hg38: chr7:69,393,633-69,405,124) in WTC11-ngn2 cells51 (i.e., WTC11 cells with a doxycycline-inducible mouse Ngn2 transgene). Briefly, WTC11-ngn2 cells were cultured in mTeSR1 media (STEMCELL Technologies) with daily media changes following normal WTC11 maintenance protocols. Cells were seeded at a density of 300k cells per 6-well in mTeSR1 media plus Rock Inhibitor (Selleckchem) and cultured for one day. WTC11-ngn2 p37+21 cells (p37= passage number before ngn2 introduction, +21= passage number after the ngn2 insertion) were then transfected with 800ng of each of the four sgRNAs (Supplementary Table 11), 6250ng of TrueCut Cas9 Protein v2 (Invitrogen), and 500ng of MSCV Puro-SV40:GFP plasmid (Addgene) using Lipofectamine CRISPRMAX Cas9 Transfection Reagent (Thermo Scientific) following the manufacturer's protocol. On the second day post transfection, cells were washed in 1X PBS, dissociated from the plate using Accutase (STEMCELL Technologies), quenched with 1X PBS, spun down and resuspended in a FACs buffer consisting of 1X PBS, 0.5M EDTA (Neta Scientific), 1M HEPES PH7.0 (Neta Scientific), 1% FBS, and Rock Inhibitor. Cells were filtered through a cell strainer, then GFP positive single cells were sorted on a BD FACSAria Flow Cytometer or equivalent using a 100-micron nozzle into 96-well plates containing mTeSR media supplemented with Rock Inhibitor, 1% Penicillin-Streptomycin (ThermoFisher), and 10% CloneR2 (STEMCELL Technologies). Individual colonies were expanded incrementally when wells became confluent. DNA was extracted from a subset of cells of each colony using AllPrep DNA/RNA Mini kit (Qiagen). To validate the deletions, gDNA was extracted using AllPrep DNA/RNA Mini kit (Qiagen), followed by genotyping of each colony using KOD One PCR Master Mix (DiagnoCine) with two unique primer sets (Supplementary Table 11). Passage 37+27 WTC11-ngn2 cell lines were differentiated into day 14 neurons following a previously described protocol. In short, cells were seeded and grown in pre-differentiation media. On the third day, cells were dissociated, counted, and plated in differentiation media according to recommended seeding densities. Cells were grown for 14 days, with only a partial media change on day 7. On day 14, DNA and RNA were extracted from the B14804-/- and wild-type cells using AllPrep DNA/RNA Mini kit (Qiagen). cDNA was synthesized from extracted RNA using SuperScript™ III Reverse Transcriptase (Invitrogen) following manufacturer's protocol. cDNA was diluted 1:10 and used for qPCR with SsoFast EvaGreen Supermix (BioRad). qPCR reactions were done in triplicate and normalized against Gapdh. Frozen B14804-/- and B14804+/+ neurons were used to generate Hi-C libraries as described above.

Timing and spatial scale

ChiP-seq, Hi-C, and Capture Hi-C data were collected starting on September 2018 and ending on May 2023. The frequency and periodicity of sampling was highly variable and dependent on availability of the samples and time from personnel working on the project.
Heart imaging data were collected in May 2022. Ear imaging data were collected between April and June 2022.

All experiments were carried out in the lab, hence spatial scale is not applicable.

Data exclusions

No data were excluded

Reproducibility

Hi-C and ChIP-seq data between biological replicates were combined after verifying their correlation based on the Pearson correlation as calculated by the HiCExplorer and deeptools packages, respectively (See Extended Data Fig. 1). Samples were highly correlated.

Randomization

Samples were grouped by species

Blinding

Blinding was not applicable for the majority of the study because we were not expecting any detection bias.
Blinding could have been applied when comparing the heart tissues from mice with the CRISPR deletion and the wild-type mice. However, the person performing the analysis (Dr. Sandra Rugonyi) was not blinded as all the images were given to her annotated. Nevertheless, the quantitative analysis was unbiased. She took 'samples' from 3-4 regions in the image (trying to keep corresponding locations - that is to sample approximately the same regions in all hearts) and then performed the analysis averaging results from each heart and then aggregating the results by group. Also, for quantitative results we did not exclude any hearts.

Did the study involve field work? ☐ Yes ☒ No

Reporting for specific materials, systems and methods

We require information from authors about some types of materials, experimental systems and methods used in many studies. Here, indicate whether each material, system or method listed is relevant to your study. If you are not sure if a list item applies to your research, read the appropriate section before selecting a response.

Materials & experimental systems

n/a	Involved in the study	
☐	☒	Antibodies
☐	☒	Eukaryotic cell lines
☒	☐	Palaeontology and archaeology
☐	☒	Animals and other organisms
☒	☐	Clinical data
☒	☐	Dual use research of concern
☒	☐	Plants

Methods

n/a	Involved in the study	
☐	☒	ChIP-seq
☒	☐	Flow cytometry
☒	☐	MRI-based neuroimaging

Antibodies

Antibodies used

Only primary antibodies were used. The information is reported below.

H3K4me1 (ab8895, Abcam, https://www.abcam.com/products/primary-antibodies/histone-h3-mono-methyl-k4-antibody-chip-grade-ab8895.html). Suitable for: ICC/IF, ChIP, WB, IHC-P. Reacts with: Mouse, Rat, Cow, Human. Isotype: IgG.

H3K4me3 (ab8580, Abcam, https://www.abcam.com/products/primary-antibodies/histone-h3-tri-methyl-k4-antibody-chip-grade-ab8580.html), Rabbit polyclonal to Histone H3 (tri methyl K4) - ChIP Grade; Suitable for: PepArr, ChIP, WB, IHC-P, ICC/IF; Reacts with: Cow, Human; Isotype: IgG.

H3K27ac (ab4729, Abcam, https://www.abcam.com/products/primary-antibodies/histone-h3-acetyl-k27-antibody-chip-grade-ab4729.html); Rabbit polyclonal to Histone H3 (acetyl K27) - ChIP Grade; Suitable for: ICC/IF, WB, IHC-P, ChIP, PepArr; Reacts with: Mouse, Rat, Cow, Human, Recombinant fragment; Isotype: IgG.

H3K27me3 (39155, Active Motif, https://www.activemotif.com/catalog/details/39155); ChIP Grade; Suitable for: Cut&Tag, CHiP-seq, Western Blot, IF, ICC; Reacts Human, Mouse, Wide Range Predicted; Isotype: IgG.

CTCF (3418s, Cell Signaling Technologies, https://www.cellsignal.com/products/primary-antibodies/ctcf-d31h2-xp-rabbit-mab/3418). Reacts with: Mouse, Rat, Monkey, Human. Isotype: Rabbit IgG.

(1:400) Alexa Fluor donkey anti-rabbit 488
Invitrogen / Thermo Fisher Scientific
Cat Num A21206
RRID:AB_2535792

(1:400) Alexa Fluor donkey anti-rabbit 546
Invitrogen / Thermo Fisher Scientific
Cat Num A10040
RRID:AB_2534016

(1:400) Alexa Fluor donkey anti-rabbit 647
Invitrogen / Thermo Fisher Scientific
Cat Num A31573
RRID:AB_2536183

(1:100) Alexa Fluor™ 647 Phalloidin
Invitrogen / Thermo Fisher Scientific
Cat Num A22287
RRID:AB_2620155

Validation

H3K4me1 (ab8895): Tested for ChIP (from Abcam): Use 2 µg for 25 µg of chromatin. We recommend Myo-D ChIP primer pair ab269261 as positive control.

H3K4me3 (ab8580). Tested for ChIP (from Abcam): Use 2 µg for 25 µg of chromatin. We recommend GAPDH positive control ChIP primer pair ab267832 as positive control).

H3K27ac (ab4729). Tested for ChIP (from Abcam): Use 2 µg for 25 µg of chromatin. We recommend GAPDH positive control ChIP primer pair ab267832 as a positive control.

H3K27me3 (39155, Active Motif). Validated by Active Motif using 5 µg each. There are 539 references including ChIP-seq with this antibody.

CTCF (3418s, Cell Signaling Technologies). Tested for ChIP (from CST): Chromatin immunoprecipitations were performed with cross-linked chromatin from Hela cells and CTCF (D31H2) XP® Rabbit mAb, using SimpleChIP® Enzymatic Chromatin IP Kit (Magnetic Beads) #9003. DNA Libraries were prepared using DNA Library Prep Kit for Illumina® (ChIP-seq, CUT&RUN) #56795.

Statement on antibody validation from Invitrogen:
Invitrogen antibodies are currently undergoing a rigorous two-part testing approach: Part 1, Target specificity verification; Part 2,

Functional application validation. More details:
https://www.thermofisher.com/us/en/home/life-science/antibodies/invitrogen-antibody-validation.html

(1:1000) Myosin7a
Proteus Biosciences
Cat Num 25-6790
RRID: AB_10015251
Waltham, MA (previously in Ramona, CA)
One of the most commonly used antibodies in the inner ear field to detect hair cells in multiple species. Here's one recent example showing this specific myo7a antibody staining in a newly created KO mouse of Myo7a:
Myosin-VIIa is expressed in multiple isoforms and essential for tensioning the hair cell mechanotransduction complex
Li et al., 2020
Nat Communications
PMID: 32350269

Eukaryotic cell lines

Policy information about cell lines and Sex and Gender in Research

Cell line source(s)	Lymphoblastoid cell lines (LCLs) for Nomascus and Hylobates were generated in the Carbone Lab. Sex information for all subjects is reported in Supplementary Table 2. WCT11-ngn2 were provided by Dr. Li Gan (Gladstone Institute)
Authentication	The cell lines used were not authenticated
Mycoplasma contamination	No mycoplasma contamination was ever found
Commonly misidentified lines (See ICLAC register)	No commonly misidentified cell lines were used in the study

Animals and other research organisms

Policy information about studies involving animals; ARRIVE guidelines recommended for reporting animal research, and Sex and Gender in Research

Laboratory animals	FVB/NJ (Jackson Lab; 001800) mice were used for the CRISPR experiment. The hearts were dissected out at the age of 8 months for H&E staining, P4 for qPCR, and P5 for Capture Hi-C. Mice were housed at room temperature with humidity 40-60%.
Wild animals	No wild animals were used for this study
Reporting on sex	We used one male individual and one female individual for each of the species. Sex-based analyses were not done in this study as they were not relevant. Data was obtained on each sex separately and then pooled for the final analyses. No sex effects were observed.
Field-collected samples	No field collected samples were used in the study
Ethics oversight	All mouse work was approved by the UCSF Institutional Animal Care and Use Program (IACUC) protocol number AN197608 and was conducted in accordance with AALAC and NIH guidelines.

Note that full information on the approval of the study protocol must also be provided in the manuscript.

Plants

Seed stocks	N/A
Novel plant genotypes	N/A
Authentication	N/A

ChIP-seq

Data deposition

☒ Confirm that both raw and final processed data have been deposited in a public database such as GEO.

☒ Confirm that you have deposited or provided access to graph files (e.g. BED files) for the called peaks.

Data access links *May remain private before publication.*	GSE197926: https://www.ncbi.nlm.nih.gov/geo/query/acc.cgi?acc=GSE197926

Files in database submission	The GEO entry contains all the raw and processed HiC, ChIP-seq, Capture HiC data generated in this study, from human, gibbons, rhesus, mouse, caroli and pahari mouse and rat.
Genome browser session (e.g. UCSC)	N/A

Methodology

Replicates	Replicate information is reported in the manuscript and supplementary table 2
Sequencing depth	Sequencing depths and statistics are reported in Supplementary Table 2
Antibodies	H3K4me1 (ab8895, Abcam), H3K4me3 (ab8580, Abcam), H3K27ac (ab4729, Abcam), H3K27me3 (39155, Active Motif), CTCF (3418s, Cell Signaling Technologies)
Peak calling parameters	peaks were only called for CTCF data:<br>macs2 callpeak -B -t $chip -c $control -f BAM -g $genome --nomodel --extsize 300<br><br>For all the other marks, not called but other graphical data such as fold enrichment pile ups were calculated as follows:<br>macs2 callpeak -B -t $chip -c $control -f BAM -g $genome --nomodel --extsize 300<br>macs2 bdgcmp -t $treatment -c $background -m FE
Data quality	Raw data was QC'd used FastQC, agreement (pearson correlation) between replicates and among marks/input was used to ensure ChIP success
Software	The following software were used: macs2 2.2.5, Bowtie2 2.3.2,deeptools 3.4.3, bedtools v2.30.0, samtools 1.3.1, ChromHMM 1.20,
